# Cell density alters bacterial community structure in culture-enriched 16S rRNA gene microbiota profiling

**DOI:** 10.1186/s13104-020-05113-2

**Published:** 2020-06-03

**Authors:** Bishnu Adhikari, Young Min Kwon

**Affiliations:** 1 Department of Poultry Science, College of Agricultural, Food and Life Sciences, University of Arkansas, Fayetteville, AR 72701 USA; 2 Cell and Molecular Biology Program, University of Arkansas, Fayetteville, AR 72701 USA

**Keywords:** Microbiota, Culture-enriched, Cell density, 16S rRNA gene sequencing, MRS agar

## Abstract

**Objective:**

Microbial community profiling using 16S rRNA gene has provided invaluable insights into diverse microbial communities. Recently a few studies have attempted to use 16S rRNA gene microbiota profiling in combination with the conventional culture methods to explore bacterial communities. In this “culture-enriched microbiota profiling” approach, microbes in a sample are cultured on solid media, and the resulting colonies are combined and subjected to 16S rRNA gene microbiota profiling. Here we investigated the effect of cell densities as determined by varying levels of sample dilution on the culture-enriched microbiota profiles using De Man, Rogosa and Sharpe (MRS) agar medium as a model system.

**Results:**

Cecal samples collected from 10 healthy chickens were serially diluted to 10^2^ fold (M-LOW), 10^4^ fold (M-MEDIUM), and 10^6^ fold (M-HIGH), and the dilutions were plated on MRS agar. 16S rRNA gene profiling showed that the relative abundance of certain genera showed gradual increase (*Pediococcus* and *Enterococcus*) or decrease (*Lactobacillus* and *Turicibacter*) with higher dilutions, though it was significant only for *Pediococcus* (p < 0.05). The result indicates that the dilution levels of the samples can alter the resulting microbiota profiles via unknown density-dependent mechanisms and thus should be considered for designing experiments using culture-enriched microbiota profiling.

## Introduction

One of the goals of exploring gut microbiota in food-producing animals is to exploit the abundant bioresources in gut environment to promote gut health, control of enteric diseases and thus overall growth performance of the animals [1–3]. In recent years, some limitations in 16S rRNA gene microbiota profiling approach and the need for retrieval of cultured live bacteria for subsequent use for probiotic applications have created a new approach combining culture-independent microbiota profiling approach with conventional culture methods [4, 5]. This new branch in microbiomics, called “culture-enriched microbiota profiling”, attempts to use the culture methods to grow live microbes, which are then further analyzed by 16S gene microbiota profiling [6].

In the study by Sibley et al. (2011), the authors directly evaluated the cultivability of the airway microbiota by analyzing samples from cystic fibrosis patients using culture-enriched molecular profiling approach. The results demonstrated that combining culture-dependent and culture-independent approaches enhances the sensitivity of either approach alone. In a more recent study by Lau et al. (2016), the similar approach was used to investigate the fecal microbiotas that were readily recovered on culture media [7]. They demonstrated that the majority of OTUs (Operational taxonomic unit) detected from metagenomic DNA could be detected through culture-enriched molecular profiling, and culture-enriched profiling detected greater diversity than culture-independent method [7]. The utility of the culture-enriched molecular profiling was further demonstrated by successful target culturing of the family *Lachnospiraceae* from the specific growth conditions where this family was significantly enriched [7].

In the study by Brown et al. [8], the culture-enriched molecular profiles of human fecal microbiota were compared with direct 16S rRNA gene profiles, and there was a statistically significant correlation between the two types of profiles at the species level [8]. Our group also analyzed the bacterial populations recovered on MRS agar by 16S rRNA gene profiling to compare lactic acid bacterial populations in different regions of chicken gut [9].

In the current study we sought to explore the experimental variables that might have influence on the microbiota profiles obtained from culture-enriched bacterial populations. Specifically, we were interested in the importance of cell density as determined by dilution levels of the microbiota samples in assessing the structure of culture-recovered bacterial populations. We used MRS agar medium as a simple model system to study the role of the dilution factor in the composition and structure of MRS-recovered bacterial populations originated from chicken cecal contents.

## Main text

### Materials and methods

Ten breeder hens of 32 weeks old were humanely euthanized by CO_2_ inhalation, and one whole cecum from each hen was collected aseptically. The cecal contents were removed, serially diluted with 1X PBS to 10^2^ fold (M-LOW), 10^4^ fold (M-MEDIUM), and 10^6^ fold (M-HIGH) dilutions. These dilutions were plated on MRS agar plates and incubated for 24 h under a microaerophilic condition at 37 °C. The average log_10_ colonies forming units (CFUs) per ceca recovered on MRS plates was 9.84 ± 0.157 (mean ± standard error). There were on average 125 ± 27.76 CFUs/plate on M-HIGH group for 10 cecal samples. Genomic DNA was extracted from both cecal contents (T-ZERO) and bacterial cells recovered from MRS agar plates using QIAamp DNA Mini Kit, Qiagen. Thus, altogether 40 DNA samples were used to amplify V1–V3 region of 16S rRNA gene using barcode-tagged universal primers: 27F (5′-AGRGTTYGATYMTGGCTCAG-3′) and 533R (5′-TTACCGCGGCTGCTGGCAC-3′) with attached Illumina adapters as described previously [9, 10]. The resulting PCR amplicons were analyzed via MiSeq sequencing with paired-end read 300 cycle option. Quantitative Insights into Microbial Ecology (QIIME) version 1.9.1 was used to analyze the MiSeq Illumina reads [11]. Reference sequences and taxonomy file from NCBI RefSeq 16S RNA sequence database were used for picking OTU and taxonomic classification [12]. OTU BIOM (biological observation matrix) table was normalized with cumulative sum scaling (CSS) method with QIIME [13], which was then used for alpha and beta diversity analyses.

### Results

**Fig. 1:**
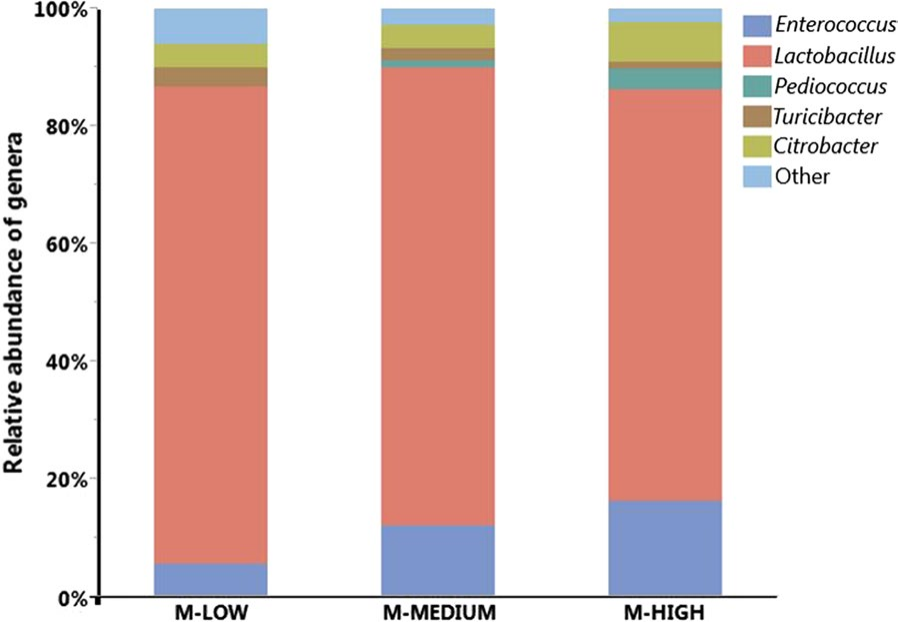
Relative abundance of major bacterial genera recovered on MRS plates from different dilutions. M-LOW, M-MEDIUM, and M-HIGH represent bacterial population recovered on MRS from 10^2^, 10^4^, and 10^6^ fold dilutions, respectively

**Fig. 2:**
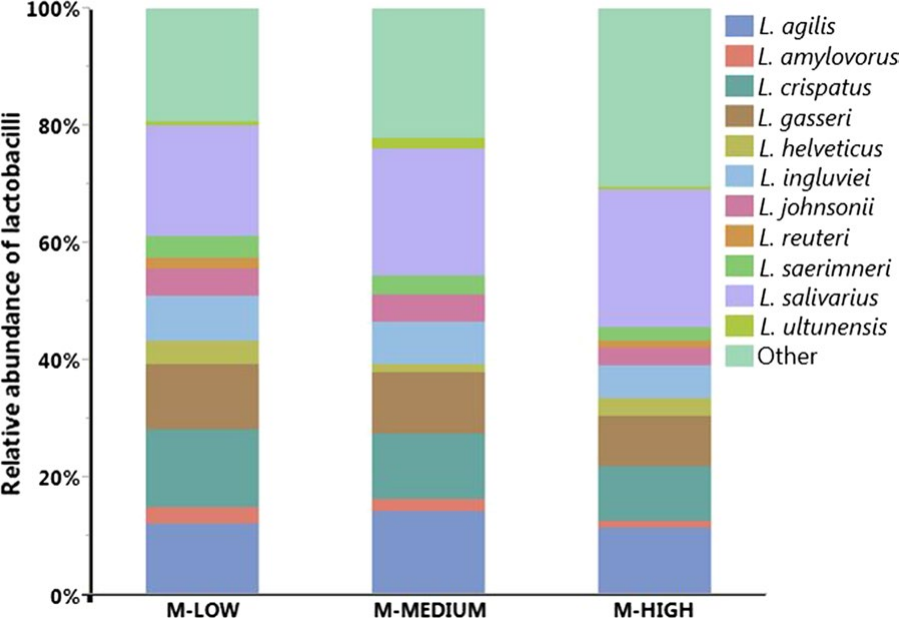
Relative abundance of major *Lactobacillus* species recovered on MRS plates from different dilutions. M-LOW, M-MEDIUM, and M-HIGH represent bacterial population recovered on MRS from 10^2^, 10^4^, and 10^6^ fold dilutions, respectively

There were total 1,707,295 reads after demultiplexing and quality filtering whose sizes ranged from 410 to 580 bp. Summarizing OTU BIOM table after removing low coverage samples (< 100) and CSS normalization resulted mean sample depth of 115.71 ± 6.93 reads per sample. Taxonomic analysis among MRS selected groups revealed that at genus level there were mainly five major genera (> 1%) recovered on MRS agar plates from three different dilutions as shown in Fig. 1. Among them, *Lactobacillus* (76.16%) was dominant genera followed by *Enterococcus* (11.59%), *Citrobacter* (4.97%), *Turicibacter* (2.03%) and *Pediococcus* (1.67%). Occurrence of different genera that do not belong to Lactic acid bacteria (LAB) suggests non-stringent selectivity of MRS agar plates, which confirms to our previous observation [9]. At species level within *Lactobacillus* species recovered on MRS agar, *L. salivarius* (21.44%) was the predominant one, followed by *L. agilis* (12.62%), *L. crispatus* (11.21%), *L. gasseri* (10.07*%), L. ingluviei* (6.77%), *L. johnsonii* (4.09%) and *L. saerimneri* (3.17%) as shown in Fig. 2. *L. salivarius* and *L. agilis* were consistently predominant across all dilutions.

The result of alpha diversity analysis as measured by observed OTUs metric showed that the alpha diversity was similar among the 3 MRS groups, while T-ZERO group had significantly higher alpha diversity as compared to the 3 MRS dilution groups (p < 0.05) (Additional file 1: Figure S1). The result agrees with the expectation, because only a subset of bacterial species in the cecal samples can grow on MRS agar medium while T-ZERO should capture all species that are represented in the extracted metagenomic DNA.

To investigate the effect of cell density in cecal samples as determined by dilution levels on the relative abundance of different taxonomic groups, we performed statistical analysis as summarized in Table 1. The relative abundance of all OTUs found in MRS groups were also determined from the directly isolated DNA samples (T-ZERO) and included in the statistical analysis as a reference for comparison. At the phylum level, there was no significant difference in the relative abundance of either Firmicutes or Proteobacteria across all 4 groups (Table 1). Although there was no statistical significance, the relative abundance of Firmicutes was consistently higher in 3 MRS groups as compared to T-ZERO, which is largely due to the enrichment of the dominant genus *Lactobacillus* on MRS agar plates. At genus level, *Turicibacter* showed the clear trend of decreasing relative abundance levels with increasing dilutions (11.8%, 3.1%, 1.9%, and 1.2% in T-ZERO, M-LOW, M-MEDIUM, and M-HIGH, respectively). In case of *Lactobacillus*, similar decreasing trend was observed with increasing dilutions among MRS groups (81.2%, 77.8%, and 70.0% in M-LOW, M-MEDIUM, and M-HIGH, respectively). On the contrary, two genera *Enterococcus* and *Pediococcus* showed increasing levels of relative abundance as the dilution increased. However, statistical difference was observed only with *Pediococcus* across the different groups (p < 0.05). Interestingly, no *Pediococcus* was found in both T-ZERO and M-LOW, while it increased to 1.3% (M-MEDIUM) and 3.5% (M-HIGH) with higher dilutions. In addition to *Pediococcus*, other genera such as *Streptococcus* and *Bacillus* also were not recovered from T-ZERO, while recovered on MRS groups. When the relative abundance of all LAB (*Enterococcus*, *Pediococcus*, and *Streptococcus*) excluding genus *Lactobacillus* was compared, it showed consistently increasing trends as the dilution increased (p < 0.05). On the contrary, *L. reuteri* present at 6.8% in T-ZERO was significantly lower or not detected among MRS groups (< 0.05).

**Table 1:** Summary of the relative abundance levels of different taxonomic groups

Level	Taxa	T-ZERO (%)	M-LOW (%)	M-MEDIUM (%)	M-HIGH (%)
Phylum	Firmicutes	(82.03 ± 7.52)^a^	(93.35 ± 3.82)^a^	(94.91 ± 3.97)^a^	(91.68 ± 5.84)^a^
	Proteobacteria	(17.97 ± 7.52)^a^	(6.12 ± 3.33)^a^	(5.09 ± 3.97)^a^	(7.98 ± 5.85)^a^
Genus	Lactic acid bacteria (LAB)				
	*Lactobacillus*	(69.01 ± 6.15)^a^	(81.21 ± 4.48)^a^	(77.82 ± 5.27)^a^	(70.01 ± 5.07)^a^
	*Enterococcus*	(1.24 ± 1.24)^b^	(5.62 ± 2.31)^ab^	(12.11 ± 3.66)^a^	16.37 ± 5.64)^a^
	*Pediococcus*	(0.00 ± 0.00)^b^	(0.00 ± 0.00)^b^	(1.31 ± 0.67)^ab^	(3.50 ± 1.47)^a^
	*Streptococcus*	(0.00 ± 0.00)^a^	(1.78 ± 1.22)^a^	(0.49 ± 0.49)^a^	(0.66 ± 0.66)^a^
	Other than LAB				
	*Bacillus*	(0.00 ± 0.00)^a^	(0.59 ± 0.59)^a^	(0.00 ± 0.00)^a^	(0.00 ± 0.00)^a^
	*Turicibacter*	(11.76 ± 1.72)^a^	(3.11 ± 1.85)^b^	(1.93 ± 0.99)^b^	(1.16 ± 0.77)^b^
	*Citrobacter*	(1.69 ± 1.69)^a^	(4.03 ± 3.40)^a^	(4.03 ± 4.03)^a^	(6.74 ± 5.96)^a^
	Other grouping				
	Non *Lactobacillus*	(30.99 ± 6.15)^a^	(18.79 ± 4.48)^a^	(22.18 ± 5.27)^a^	(29.99 ± 5.07)^a^
	LAB other than Lactobacillus*	(1.24 ± 1.24)^c^	(7.99 ± 2.98)^bc^	(13.91 ± 4.08)^ab^	(20.52 ± 5.24)^a^
	Other than LAB	(29.74 ± 6.00)^a^	(10.80 ± 4.80)^b^	(8.27 ± 3.90)^b^	(9.47 ± 5.76)^b^
Species	*L. johnsonii*	(0.00 ± 0.00)^b^	(4.66 ± 1.44)^a^	(4.56 ± 1.23)^a^	(3.12 ± 1.39)^ab^
	*L. reuteri*	(6.76 ± 1.77)^a^	(1.70 ± 0.84)^b^	(0.00 ± 0.00)^b^	(1.00 ± 1.00)^b^
	*L. salivarius*	(16.38 ± 1.56)^b^	(18.85 ± 1.74)^ab^	21.66 ± 2.13)^ab^	(23.53 ± 3.19)^a^
	*L. ultunensis*	(0.00 ± 0.00)^b^	(0.64 ± 0.64)^ab^	(1.72 ± 0.87)^a^	(0.54 ± 0.54)^ab^

Values are presented in mean ± SEM (Standard Errors of Means). Different letters across each row show statistically significance at *p *< 0.05 (ANOVA, Student *t* test). *L. acidophilus* only present on M-HIGH, absent in all other groups (0.33 ± 0.33)  %. Other species didn’t show any significant differences among different groups

### Discussion

Recent approaches attempting to characterize microbial community in a high-throughput manner using bacterial colonies recovered on various agar media have successfully isolated novel bacterial species and spore-formers that have escaped detection by culture-independent method alone [4, 7]. Multiple studies using culturomics approach have successfully isolated numerous novel species, which remained previously uncultured members [14–17]. On the contrary, studies in which the microbiota profiles were compared between culture-dependent and culture-independent approaches have reported that each approach captured unique subsets of microorganism [5, 18]. These studies strongly suggest that the limitation of 16S rRNA gene profiling approach can be overcome at least partially by the use of culture-enriched microbiota profiling or culturomics approaches.

In the present study, we sought to evaluate the hypothesis that the relative abundance levels of bacterial taxa in microbial communities as determined by 16S rRNA gene profiling of culture-enriched bacteria change with different levels of sample dilution. The hypothesis was built on the followings: (1) there are a number of interaction mechanisms operating among the bacterial cells in microbial community and (2) the assumption that the cell density of the samples, which in turn changes the physical distance between the cells on solid medium when plated, would influence those antagonistic interactions during formation of colonies on solid media.

In the recent studies using culture-enriched microbiota profiling, the researchers used slightly different procedures to recover the bacterial colonies to represent taxa that are recovered on a solid media in terms of the dilution levels of the original samples. For example, Browne et al. [8] plated serial dilutions of the samples, and the lowest dilutions that allowed the growth of distinct colonies on agar plates were used to collect the colonies for microbiota profiling [8]. Rettedal et al. combined multiple dilutions (2–3 consecutive dilutions) of the human fecal samples from each media in equal proportions to better represent the bacteria capable of growing on each media, and cells were typically recovered from samples diluted 100,000- to 1,000,000-fold [5].

The results in this study demonstrated that the levels of dilution of the chicken cecal samples plated on MRS plates changed the resulting microbiota profiles in a dilution level-dependent manner. The changes in many taxa at genus and species levels were not random, but they followed the patterns closely associated with the level of dilution. There are multiple antagonistic mechanisms in microbial communities that govern interactions among the members, including colicins, bacteriocins, contact-dependent growth inhibition systems, and type VI secretion systems [19]. Quorum sensing is also an important mechanism influencing microbial community in a cell density-dependent manner [20]. In case of *Lactobacillus*, the bacteria acidify their environment during their metabolism and fermentation, restricting growth of other bacteria in the surroundings [21]. One of the clear trends observed was that the relative abundance of the genus *Lactobacillus* decreased consistently as the dilution increased, indicating the presence of concentration-dependent inhibition mechanism by *Lactobacillus* against non-*Lactobacillus* (Table 1). However, closer examination at species level revealed that the responses are species-dependent. The result in Table 1 shows that the different *Lactobacillu*s species displayed different patterns of relative abundance in relation to varying levels of sample dilution. For example, unlike other *Lactobacillus* species, *L. reuteri* was 6.7% in T-ZERO but was reduced significantly in all MRS-dilution groups (p < 0.05). On the contrary, *L. johnsonii* and *L. ultunensis*, which were not detected in T-ZERO, became detectable in MRS-groups at various levels. It was interesting to observe that some other genera such as *Pediococcus*, *Streptococcus*, and *Bacillus* were detected only in MRS groups, while undetected in T-ZERO.

Since the antagonistic action would be more effective in a close physical distance, the colony growth on the plates with the samples of high cell density would be altered by the inhibition mechanisms. On the contrary, when the samples are diluted to an appropriate level the inhibitory effects would be reduced significantly or completely disappeared, leading to unhindered growth of all colonies. This line of reasoning suggests that the microbiota profiles from the samples highly diluted would resemble the profiles of the direct profiling more closely. However, the result shown in Additional file 1: Figure S2 does not support this hypothesis in a clear way. It might be possible that the samples in M-LOW (10^2^-fold diluted) were already diluted sufficiently to allow unhindered growth of the colonies. Overall, the results of this study suggest that dilution factors should be considered carefully for future studies using culture-enriched microbiota profiling approach.

## Limitations

This study was conducted in a small scale using only MRS media as a model system. Therefore, it remains to be tested if similar concentration-dependent changes of culture-enriched microbiota profiles would happen when different microbiota samples and culture conditions (e.g. media and gas atmosphere) are used.

## Supplementary information

**Additional file 1: Figure S1.** Alpha diversity of the different groups as measured by observed OTUs. Bars with different letters represent statistically significant at *p *< 0.05. T-ZERO represent total bacterial populations recovered directly from cecal contents whereas M-LOW, M-MEDIUM, and M-HIGH represent bacterial population recovered on MRS from 10^2^, 10^4^, and 10^6^ fold dilutions respectively.

**Additional file 2: Figure S2.** PCoA plot showing the distances among total bacteria (T-ZERO) and MRS-selected dilution groups (M-LOW, M-MEDIUM, and M-HIGH) based on Weighted UniFrac distance metric. For T-ZERO in this analysis, only the OTUs in T-ZERO that were also found in MRS-dilution groups were used.

## Data Availability

The datasets analyzed during the current study are available from the corresponding author on reasonable request.

## Abbreviations

MRS: De Man, Rogosa and Sharpe
CFU: Colony Forming Unit
QIIME: Quantitative Insights into Microbial Ecology
OTU: Operational Taxonomic Unit
BIOM: Biological Observation Matrix
CSS: Cumulative Sum Scaling
