# Supplementary material for: Cell density alters bacterial community structure in culture-enriched 16S rRNA gene microbiota profiling
Source: BMC Res Notes. 2020 Jun 3;13:269. doi: 10.1186/s13104-020-05113-2 (PMC7268277; doi:10.1186/s13104-020-05113-2)

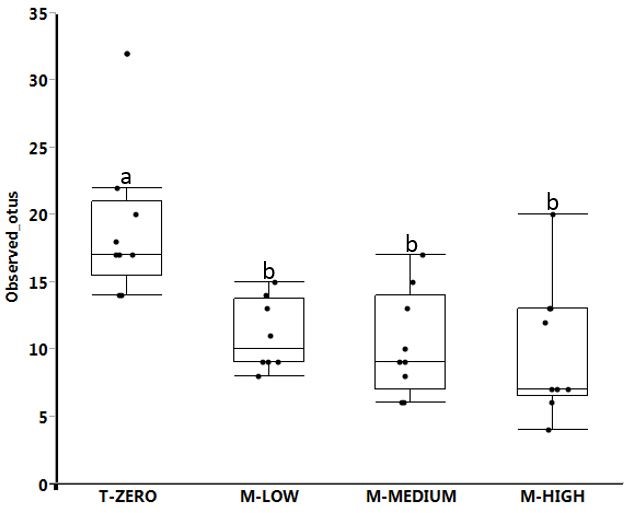
**Supplementary Figure 1.** Alpha diversity of the different groups as measured by observed OTUs. Bars with different letters represent statistically significant at *p*<0.05. T-ZERO represent total bacterial populations recovered directly from cecal contents whereas M-LOW, M-MEDIUM, and M-HIGH represent bacterial population recovered on MRS from 10^2^, 10^4^, and 10^6^ fold dilutions respectively.

**Supplementary Figure 2.**  PCoA plot showing the distances among total bacteria (T-ZERO) and MRS-selected dilution groups (M-LOW, M-MEDIUM, and M-HIGH) based on Weighted UniFrac distance metric. For T-ZERO in this analysis, only the OTUs in T-ZERO that were also found in MRS-dilution groups were used.


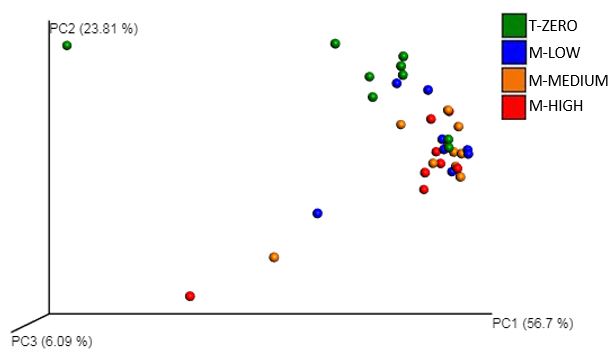

Supplement: Supplementary file 1 — Additional file 1: Figure S1. Alpha diversity of the different groups as measured by observed OTUs. Bars with different letters represent statistically significant at p < 0.05. T-ZERO represent total bacterial populations recovered directly from cecal contents whereas M-LOW, M-MEDIUM, and M-HIGH represent bacterial population recovered on MRS from 102, 104, and 106 fold dilutions respectively. [file 13104_2020_5113_MOESM1_ESM.docx]
