# Supplementary material for: Cell density alters bacterial community structure in culture-enriched 16S rRNA gene microbiota profiling
Source: BMC Res Notes. 2020 Jun 3;13:269. doi: 10.1186/s13104-020-05113-2 (PMC7268277; doi:10.1186/s13104-020-05113-2)

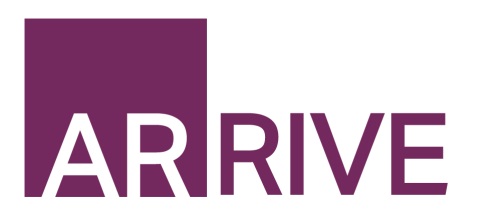


The ARRIVE Guidelines Checklist

Animal Research: Reporting In Vivo Experiments

Carol Kilkenny^1^, William J Browne^2^, Innes C Cuthill^3^, Michael Emerson^4^ and Douglas G Altman^5^

*^1^The National Centre for the Replacement, Refinement and Reduction of Animals in Research, London, UK, ^2^School of Veterinary Science, University of Bristol, Bristol, UK, ^3^School of Biological Sciences, University of Bristol, Bristol, UK, ^4^National Heart and Lung Institute, Imperial College London, UK, ^5^Centre for Statistics in Medicine, University of Oxford, Oxford, UK.*

|  | | ITEM | RECOMMENDATION | Section/ Paragraph |
| --- | --- | --- | --- | --- |
| 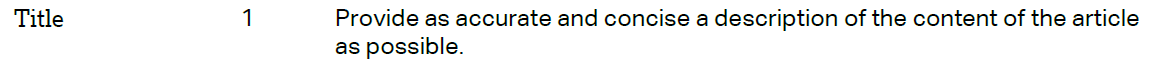 | | | Title |  |
| 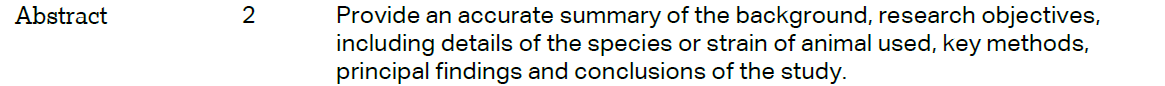 | | | Abstract |  |
| INTRODUCTION | | |  |  |
| 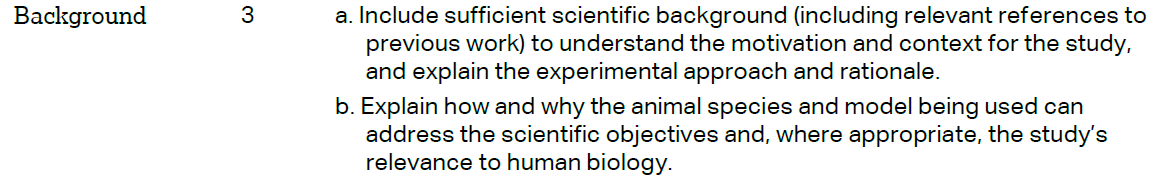 | | | Paragraphs 1-3  Paragraph 4 |  |
| 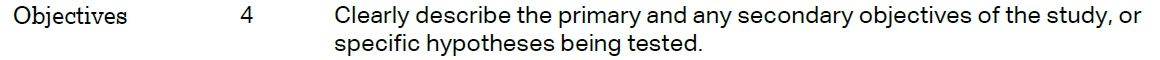 | | | Paragraph 4 |  |
| METHODS | | |  |  |
| 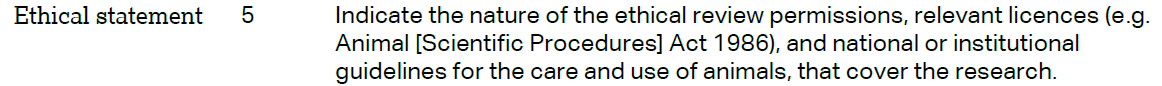 | | | Declarations section (page 10) |  |
| 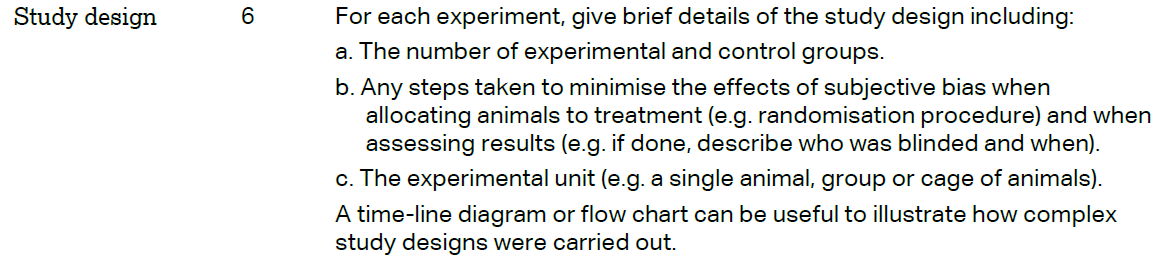 | | | Paragraph 1 |  |
| 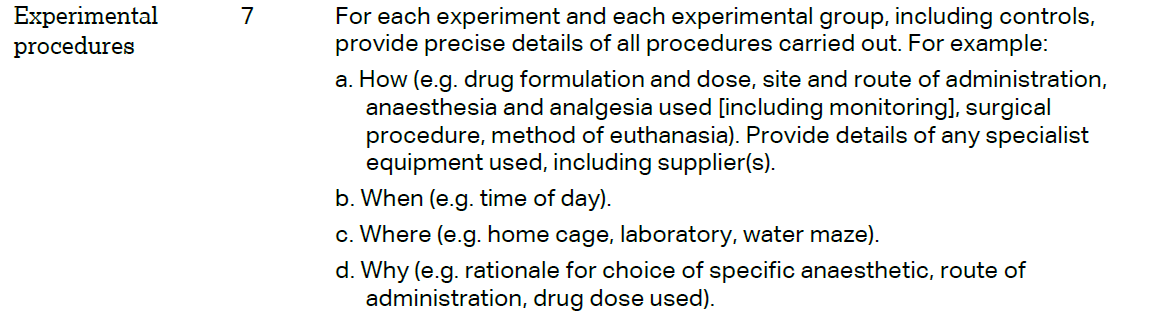 | | | Paragraph 1 |  |
| 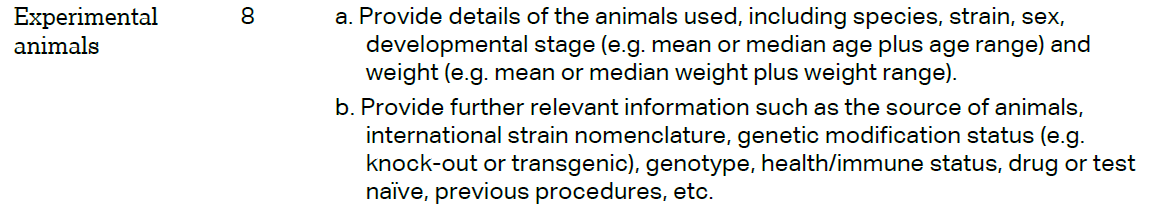 | | | Paragraph 1 |  |

The ARRIVE guidelines. Originally published in *PLoS Biology*, June 2010^1^

| 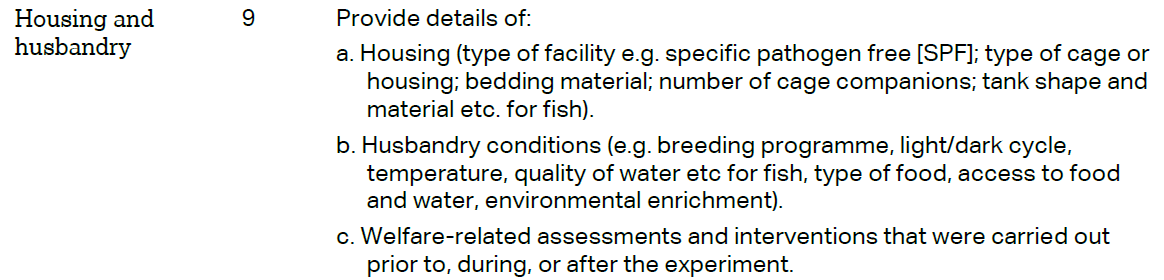 | N/A | |
| --- | --- | --- |
| 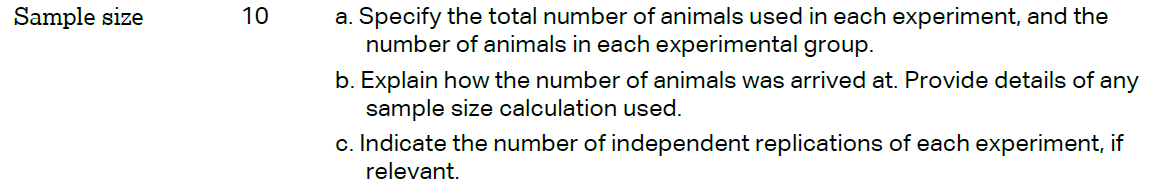 | Paragraph 1 | |
| 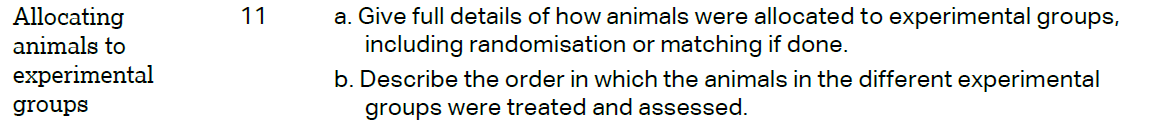 | N/A | |
| 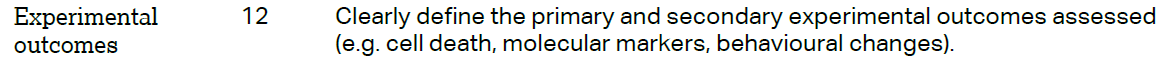 | N/A | |
| 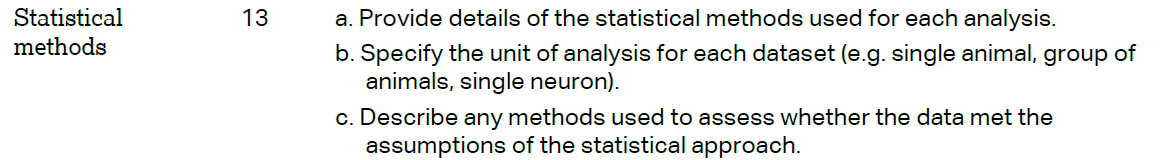 | Paragraph 1 | |
| RESULTS |  | |
| 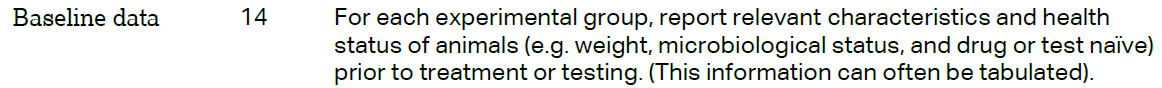 | N/A | |
| 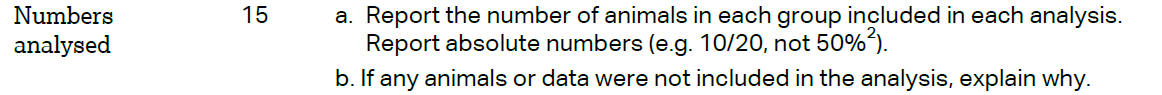 | N/A | |
| 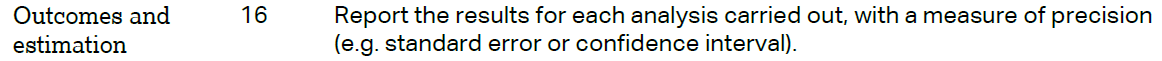 | Paragraph 1 | |
| 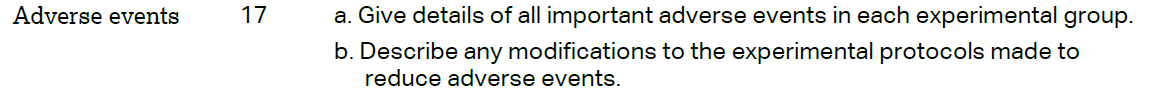 | N/A | |
| DISCUSSION |  | |
| 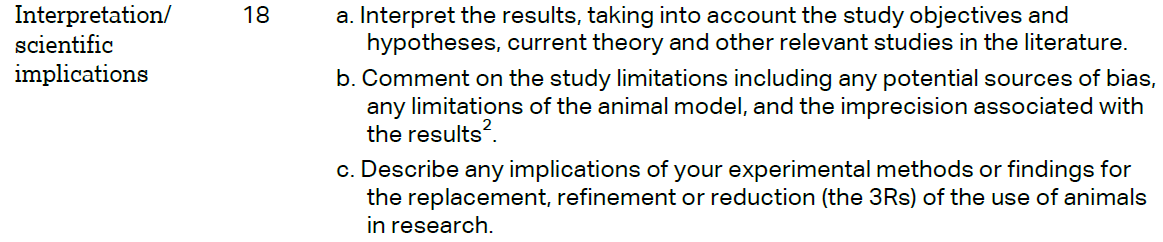 | Paragraphs 4-5  Limitations section  N/A | |
| 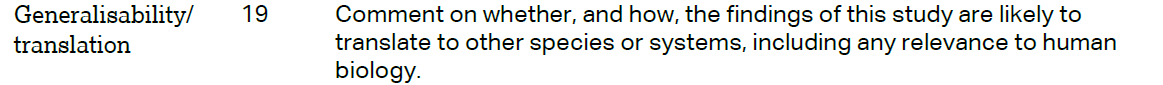 | Paragraph 5 | |
| 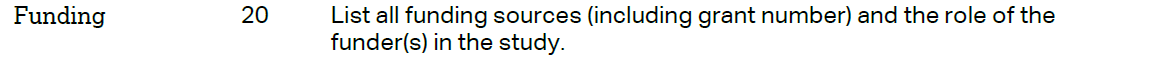 | | Page 10 |


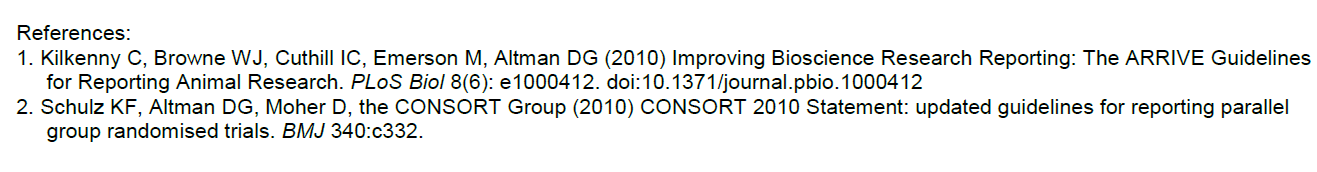

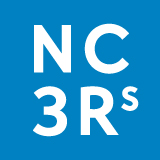

Supplement: Supplementary file 2 — Additional file 2: Figure S2. PCoA plot showing the distances among total bacteria (T-ZERO) and MRS-selected dilution groups (M-LOW, M-MEDIUM, and M-HIGH) based on Weighted UniFrac distance metric. For T-ZERO in this analysis, only the OTUs in T-ZERO that were also found in MRS-dilution groups were used. [file 13104_2020_5113_MOESM2_ESM.docx]
